# Ethyl 3-bromo-4-cyano-5-[(2-eth­oxy-2-oxoeth­yl)sulfan­yl]thio­phene-2-carboxyl­ate

**DOI:** 10.1107/S1600536813010611

**Published:** 2013-05-11

**Authors:** Xiuping Li, Xiaochuan Jia, Jing Li

**Affiliations:** a Tianjin Entry–Exit Inspection and Quarantine Bureau, Youyi road No. 33, Tianjin, People’s Republic of China

## Abstract

The title compound, C_12_H_12_BrNO_4_S_2_, was obtained by the Sandmeyer reaction from ethyl 3-amino-4-cyano-5-[(2-eth­oxy-2-oxoeth­yl)sulfan­yl]thio­phene-2-carboxyl­ate. The dihedral angle between the thiophene ring and linked CO_2_ ester group is 2.0 (5)°.

## Related literature

For background literature on the use of 3-amino-4-cyano-5-eth­oxy­carbonyl­methyl­sulfanyl-thio­phene-2-carb­oxy­lic acid ethyl ester as an important inter­mediate compound for the synthesis of thieno­pyrimidine derivatives, which are thought to be potential biologically active compounds or pharmaceuticals, see: Liu *et al.* (2008 ▶). For a related compound, see: Padmavathi *et al.* (2011 ▶).
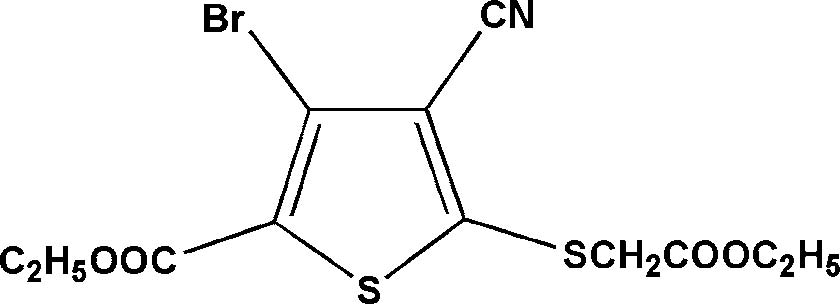


## Experimental

### 

#### Crystal data

C_12_H_12_BrNO_4_S_2_
*M* *_r_* = 378.26
Monoclinic, 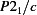

*a* = 8.5896 (17) Å
*b* = 10.837 (2) Å
*c* = 16.584 (3) Å
β = 99.44 (3)°
*V* = 1522.8 (5) Å^3^
*Z* = 4
Mo *K*α radiation
μ = 2.98 mm^−1^
*T* = 293 K
0.20 × 0.18 × 0.12 mm

#### Data collection

Rigaku Saturn diffractometer
Absorption correction: multi-scan (*CrystalClear*; Rigaku, 2005 ▶) *T*_min_ = 0.587, *T*_max_ = 0.716
15211 measured reflections
3596 independent reflections
2224 reflections with *I* > 2σ(*I*)
*R*_int_ = 0.067

#### Refinement

*R*[*F* ^2^ > 2σ(*F* ^2^)] = 0.052
*wR*(*F* ^2^) = 0.157
*S* = 1.00
3596 reflections
184 parameters
H-atom parameters constrained
Δρ_max_ = 0.43 e Å^−3^
Δρ_min_ = −0.71 e Å^−3^

### 

Data collection: *CrystalClear* (Rigaku, 2005 ▶); cell refinement: *CrystalClear*; data reduction: *CrystalClear*; program(s) used to solve structure: *SHELXS97* (Sheldrick, 2008 ▶); program(s) used to refine structure: *SHELXL97* (Sheldrick, 2008 ▶); molecular graphics: *SHELXTL* (Sheldrick, 2008 ▶); software used to prepare material for publication: *SHELXTL*.

## Supplementary Material

Crystal structure: contains datablock(s) global, I. DOI: 10.1107/S1600536813010611/zj2102sup1.cif

Structure factors: contains datablock(s) I. DOI: 10.1107/S1600536813010611/zj2102Isup2.hkl

Supplementary material file. DOI: 10.1107/S1600536813010611/zj2102Isup3.cml

Additional supplementary materials:  crystallographic information; 3D view; checkCIF report

## Figures and Tables

**Fig. 1.**
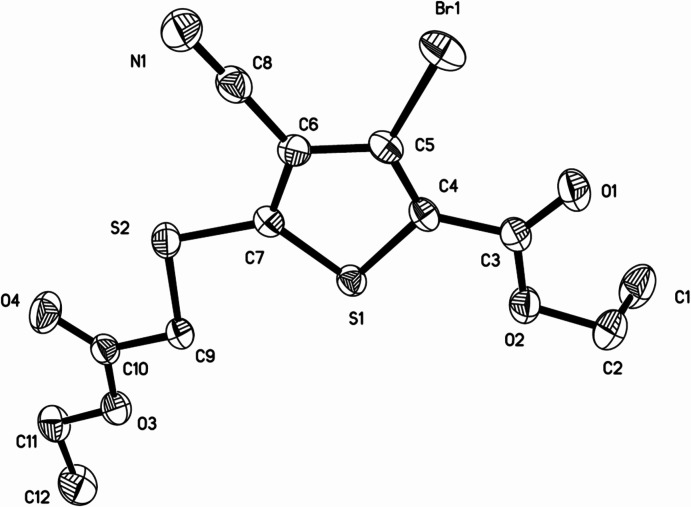
The structure of the title compound showing 30% probability ellipsoids.

**Fig. 2.**
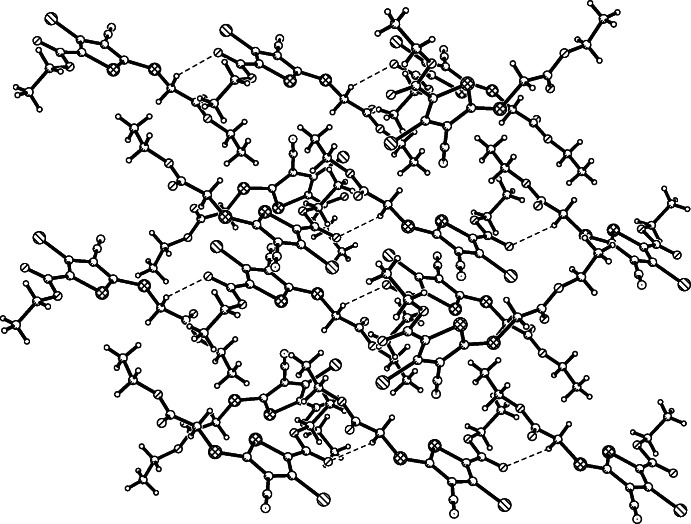
Packing structure of the title compound.

## supplementary crystallographic information

### Comment

3-Amino-4-cyano-5-ethoxycarbonylmethylsulfanyl-thiophene-2-carboxylic acid ethyl ester is an important intermediate compound for sythesis of thienothienopyrimidines derivatives, which are thought to be potential biological active compounds or pharmaceuticals (Liu, *et al.*,2008). We obtained the title compound by the Sandmeyer reaction from compound 3-Amino-4-cyano-5-ethoxycarbonylmethylsulfanyl-thiophene-2-carboxylic acid ethyl ester.The crystal for X-ray crystal structure analysis was obtained by recrystallizing the title compound in petroleum ether. In the crystal, the thiophene ring together with its four adjoint groups, *i.e.* CN, Br, S—CH_2_ and COO–,was located at one perfect plane, which is consistent with the crystal of 3-Amino-4-cyano-5-ethoxycarbonylmethylsulfany- thiophene-2-carboxylic acid ethyl ester reported in the literature (Padmavathi *et al.*, 2011). The title compound cyrstal demonstrated a crystal system of monoclinic and a spce group of P2(1)/c. There existed hydrogen bond with length of 2.554 Å between one of –SCH_2_ H atom and the O atom of carbonyl adjoined with thiophene ring of neighbor molecule.

### Experimental

To a solution of 3-Amino-4-cyano-5-ethoxycarbonylmethylsulfanyl -thiophene-2-carboxylic acid ethyl ester (1.57 g, 5 mmol) in 70% H_2_SO_4_ (13 mL) was added NaNO_2_ (0.4 g,5,7 mmol) in 5 minutes under ice water temperature. After addition, the solution was stirred for 30 min at room temperature. Then, the reaction mixture was transfered to HBr solution containing CuBr (1 g, 7 mmol). After standing overnight, water (100 mL) was added. The precipitate was collected by filtration and recrystallized from petroleum ether to afford the title compound as colourless crystals, yield 50%.

### Crystal data

**Table:**

C_12_H_12_BrNO_4_S_2_	*F*(000) = 760
*M**_r_* = 378.26	*D*_x_ = 1.650 Mg m^−^^3^
Monoclinic, *P*2_1_/*c*	Mo *K*α radiation, λ = 0.71073 Å
Hall symbol: -P 2ybc	Cell parameters from 3404 reflections
*a* = 8.5896 (17) Å	θ = 3.1–27.9°
*b* = 10.837 (2) Å	µ = 2.98 mm^−^^1^
*c* = 16.584 (3) Å	*T* = 293 K
β = 99.44 (3)°	Block, colorless
*V* = 1522.8 (5) Å^3^	0.20 × 0.18 × 0.12 mm
*Z* = 4	

### Data collection

**Table:**

Rigaku Saturn diffractometer	3596 independent reflections
Radiation source: rotating anode	2224 reflections with *I* > 2σ(*I*)
Confocal monochromator	*R*_int_ = 0.067
ω scans	θ_max_ = 27.9°, θ_min_ = 3.1°
Absorption correction: multi-scan (*CrystalClear*; Rigaku, 2005)	*h* = −11→11
*T*_min_ = 0.587, *T*_max_ = 0.716	*k* = −14→14
15211 measured reflections	*l* = −21→21

### Refinement

**Table:**

Refinement on *F*^2^	Secondary atom site location: difference Fourier map
Least-squares matrix: full	Hydrogen site location: inferred from neighbouring sites
*R*[*F*^2^ > 2σ(*F*^2^)] = 0.052	H-atom parameters constrained
*wR*(*F*^2^) = 0.157	*w* = 1/[σ^2^(*F*_o_^2^) + (0.0817*P*)^2^] where *P* = (*F*_o_^2^ + 2*F*_c_^2^)/3
*S* = 1.00	(Δ/σ)_max_ = 0.003
3596 reflections	Δρ_max_ = 0.43 e Å^−^^3^
184 parameters	Δρ_min_ = −0.71 e Å^−^^3^
0 restraints	Extinction correction: *SHELXL97* (Sheldrick, 2008), Fc^*^=kFc[1+0.001xFc^2^λ^3^/sin(2θ)]^-1/4^
Primary atom site location: structure-invariant direct methods	Extinction coefficient: 0.018 (2)

### Special details

**Table:**

Geometry. All e.s.d.'s (except the e.s.d. in the dihedral angle between two l.s. planes) are estimated using the full covariance matrix. The cell e.s.d.'s are taken into account individually in the estimation of e.s.d.'s in distances, angles and torsion angles; correlations between e.s.d.'s in cell parameters are only used when they are defined by crystal symmetry. An approximate (isotropic) treatment of cell e.s.d.'s is used for estimating e.s.d.'s involving l.s. planes.
Refinement. Refinement of *F*^2^ against ALL reflections. The weighted *R*-factor *wR* and goodness of fit *S* are based on *F*^2^, conventional *R*-factors *R* are based on *F*, with *F* set to zero for negative *F*^2^. The threshold expression of *F*^2^ > σ(*F*^2^) is used only for calculating *R*-factors(gt) *etc*. and is not relevant to the choice of reflections for refinement. *R*-factors based on *F*^2^ are statistically about twice as large as those based on *F*, and *R*- factors based on ALL data will be even larger.

### Fractional atomic coordinates and isotropic or equivalent isotropic displacement parameters (Å^2^)

**Table:**

	*x*	*y*	*z*	*U*_iso_*/*U*_eq_	
Br1	1.34684 (5)	−0.05401 (5)	0.13435 (3)	0.0723 (3)	
S1	0.95059 (11)	0.19092 (9)	0.04312 (6)	0.0421 (3)	
S2	0.75972 (12)	0.13383 (10)	0.17935 (7)	0.0505 (3)	
O1	1.3519 (4)	0.1042 (3)	−0.0246 (2)	0.0703 (9)	
O2	1.1477 (3)	0.2261 (3)	−0.07630 (18)	0.0577 (8)	
O3	0.4240 (3)	0.3685 (3)	0.10494 (18)	0.0559 (8)	
O4	0.4796 (4)	0.2478 (3)	0.2147 (2)	0.0744 (10)	
N1	1.0569 (5)	−0.0869 (4)	0.2904 (3)	0.0784 (13)	
C1	1.3411 (7)	0.3617 (5)	−0.1215 (3)	0.0825 (16)	
H1A	1.2890	0.4277	−0.0980	0.124*	
H1B	1.3794	0.3912	−0.1692	0.124*	
H1C	1.4282	0.3325	−0.0823	0.124*	
C2	1.2285 (6)	0.2598 (5)	−0.1446 (3)	0.0667 (13)	
H2A	1.1510	0.2847	−0.1911	0.080*	
H2B	1.2845	0.1885	−0.1607	0.080*	
C3	1.2245 (5)	0.1481 (4)	−0.0215 (3)	0.0468 (10)	
C4	1.1325 (4)	0.1209 (3)	0.0445 (2)	0.0420 (9)	
C5	1.1667 (5)	0.0433 (3)	0.1088 (3)	0.0440 (9)	
C6	1.0474 (4)	0.0400 (3)	0.1587 (2)	0.0427 (9)	
C7	0.9215 (4)	0.1161 (3)	0.1300 (2)	0.0390 (8)	
C8	1.0542 (5)	−0.0323 (4)	0.2309 (3)	0.0551 (11)	
C9	0.6413 (4)	0.2381 (4)	0.1105 (2)	0.0473 (10)	
H9A	0.7050	0.3072	0.0979	0.057*	
H9B	0.6003	0.1960	0.0599	0.057*	
C10	0.5069 (4)	0.2838 (4)	0.1508 (3)	0.0464 (10)	
C11	0.2964 (6)	0.4242 (5)	0.1395 (3)	0.0632 (13)	
H11A	0.3373	0.4630	0.1914	0.076*	
H11B	0.2205	0.3617	0.1488	0.076*	
C12	0.2186 (6)	0.5190 (5)	0.0800 (4)	0.0903 (18)	
H12A	0.2957	0.5782	0.0691	0.135*	
H12B	0.1371	0.5603	0.1029	0.135*	
H12C	0.1733	0.4791	0.0299	0.135*	

### Atomic displacement parameters (Å^2^)

**Table:**

	*U*^11^	*U*^22^	*U*^33^	*U*^12^	*U*^13^	*U*^23^
Br1	0.0499 (4)	0.0728 (4)	0.0911 (5)	0.0186 (2)	0.0023 (3)	0.0000 (3)
S1	0.0381 (5)	0.0419 (5)	0.0472 (6)	0.0045 (4)	0.0093 (4)	0.0038 (4)
S2	0.0452 (6)	0.0526 (6)	0.0570 (7)	0.0040 (5)	0.0186 (5)	0.0114 (5)
O1	0.0539 (19)	0.088 (2)	0.075 (2)	0.0161 (18)	0.0267 (16)	0.0067 (19)
O2	0.0532 (18)	0.0659 (19)	0.0574 (18)	0.0027 (15)	0.0195 (14)	0.0111 (16)
O3	0.0464 (16)	0.0658 (19)	0.0585 (19)	0.0130 (15)	0.0177 (14)	−0.0037 (15)
O4	0.074 (2)	0.088 (2)	0.070 (2)	0.0212 (18)	0.0363 (18)	0.0164 (19)
N1	0.073 (3)	0.077 (3)	0.087 (3)	0.012 (2)	0.020 (2)	0.035 (3)
C1	0.100 (4)	0.089 (4)	0.064 (3)	−0.022 (3)	0.030 (3)	0.002 (3)
C2	0.076 (3)	0.072 (3)	0.056 (3)	−0.004 (3)	0.022 (2)	0.008 (2)
C3	0.040 (2)	0.049 (2)	0.052 (2)	−0.0011 (19)	0.0102 (18)	−0.005 (2)
C4	0.034 (2)	0.039 (2)	0.054 (2)	0.0016 (16)	0.0101 (17)	−0.0058 (18)
C5	0.036 (2)	0.041 (2)	0.054 (2)	−0.0013 (16)	0.0032 (18)	−0.0033 (18)
C6	0.041 (2)	0.039 (2)	0.047 (2)	−0.0030 (17)	0.0034 (18)	0.0045 (17)
C7	0.036 (2)	0.0346 (19)	0.046 (2)	−0.0006 (16)	0.0064 (16)	−0.0012 (16)
C8	0.045 (2)	0.051 (2)	0.070 (3)	0.006 (2)	0.011 (2)	0.011 (2)
C9	0.036 (2)	0.059 (3)	0.048 (2)	0.0034 (19)	0.0114 (18)	0.003 (2)
C10	0.039 (2)	0.053 (2)	0.049 (2)	0.0003 (19)	0.0099 (18)	−0.003 (2)
C11	0.050 (3)	0.070 (3)	0.074 (3)	0.018 (2)	0.023 (2)	−0.008 (3)
C12	0.068 (3)	0.097 (4)	0.108 (5)	0.036 (3)	0.021 (3)	0.015 (4)

### Geometric parameters (Å, º)

**Table:**

Br1—C5	1.864 (4)	C2—H2A	0.9700
S1—C7	1.707 (4)	C2—H2B	0.9700
S1—C4	1.734 (4)	C3—C4	1.480 (6)
S2—C7	1.735 (4)	C4—C5	1.352 (5)
S2—C9	1.798 (4)	C5—C6	1.418 (6)
O1—C3	1.202 (5)	C6—C7	1.381 (5)
O2—C3	1.335 (5)	C6—C8	1.425 (6)
O2—C2	1.468 (5)	C9—C10	1.509 (5)
O3—C10	1.323 (5)	C9—H9A	0.9700
O3—C11	1.449 (5)	C9—H9B	0.9700
O4—C10	1.188 (5)	C11—C12	1.504 (7)
N1—C8	1.147 (6)	C11—H11A	0.9700
C1—C2	1.477 (7)	C11—H11B	0.9700
C1—H1A	0.9600	C12—H12A	0.9600
C1—H1B	0.9600	C12—H12B	0.9600
C1—H1C	0.9600	C12—H12C	0.9600
			
C7—S1—C4	92.15 (19)	C5—C6—C8	124.7 (4)
C7—S2—C9	100.55 (18)	C6—C7—S1	111.1 (3)
C3—O2—C2	116.1 (3)	C6—C7—S2	123.1 (3)
C10—O3—C11	115.6 (3)	S1—C7—S2	125.8 (2)
C2—C1—H1A	109.5	N1—C8—C6	177.4 (5)
C2—C1—H1B	109.5	C10—C9—S2	108.6 (3)
H1A—C1—H1B	109.5	C10—C9—H9A	110.0
C2—C1—H1C	109.5	S2—C9—H9A	110.0
H1A—C1—H1C	109.5	C10—C9—H9B	110.0
H1B—C1—H1C	109.5	S2—C9—H9B	110.0
O2—C2—C1	111.0 (4)	H9A—C9—H9B	108.4
O2—C2—H2A	109.4	O4—C10—O3	125.0 (4)
C1—C2—H2A	109.4	O4—C10—C9	124.4 (4)
O2—C2—H2B	109.4	O3—C10—C9	110.6 (4)
C1—C2—H2B	109.4	O3—C11—C12	108.0 (4)
H2A—C2—H2B	108.0	O3—C11—H11A	110.1
O1—C3—O2	124.9 (4)	C12—C11—H11A	110.1
O1—C3—C4	123.6 (4)	O3—C11—H11B	110.1
O2—C3—C4	111.4 (3)	C12—C11—H11B	110.1
C5—C4—C3	129.3 (3)	H11A—C11—H11B	108.4
C5—C4—S1	111.1 (3)	C11—C12—H12A	109.5
C3—C4—S1	119.5 (3)	C11—C12—H12B	109.5
C4—C5—C6	113.1 (3)	H12A—C12—H12B	109.5
C4—C5—Br1	126.6 (3)	C11—C12—H12C	109.5
C6—C5—Br1	120.4 (3)	H12A—C12—H12C	109.5
C7—C6—C5	112.6 (3)	H12B—C12—H12C	109.5
C7—C6—C8	122.7 (4)		
			
C3—O2—C2—C1	−83.9 (5)	C5—C6—C7—S1	−0.2 (4)
C2—O2—C3—O1	−0.4 (6)	C8—C6—C7—S1	179.4 (3)
C2—O2—C3—C4	179.9 (3)	C5—C6—C7—S2	−178.6 (3)
O1—C3—C4—C5	−2.5 (7)	C8—C6—C7—S2	0.9 (6)
O2—C3—C4—C5	177.2 (4)	C4—S1—C7—C6	−0.1 (3)
O1—C3—C4—S1	179.4 (4)	C4—S1—C7—S2	178.3 (3)
O2—C3—C4—S1	−1.0 (5)	C9—S2—C7—C6	−178.3 (3)
C7—S1—C4—C5	0.4 (3)	C9—S2—C7—S1	3.5 (3)
C7—S1—C4—C3	178.9 (3)	C7—C6—C8—N1	−36 (12)
C3—C4—C5—C6	−178.9 (4)	C5—C6—C8—N1	144 (11)
S1—C4—C5—C6	−0.6 (4)	C7—S2—C9—C10	−169.7 (3)
C3—C4—C5—Br1	0.6 (6)	C11—O3—C10—O4	3.5 (6)
S1—C4—C5—Br1	178.9 (2)	C11—O3—C10—C9	−176.8 (4)
C4—C5—C6—C7	0.5 (5)	S2—C9—C10—O4	−5.4 (6)
Br1—C5—C6—C7	−179.0 (3)	S2—C9—C10—O3	174.9 (3)
C4—C5—C6—C8	−179.0 (4)	C10—O3—C11—C12	178.9 (4)
Br1—C5—C6—C8	1.5 (5)		
